# Progress on growth faltering

**DOI:** 10.1016/S2214-109X(16)30357-6

**Published:** 2017-02

**Authors:** Rosie J Crane, James A Berkley

**Affiliations:** KEMRI/Wellcome Trust Research Programme, Kilifi, Kenya; Centre for Tropical Medicine and Global Health, Nuffield Department of Medicine, University of Oxford, Oxford OX3 7FZ, UK; KEMRI/Wellcome Trust Research Programme, Kilifi, Kenya; Centre for Tropical Medicine and Global Health, Nuffield Department of Medicine, University of Oxford, Oxford OX3 7FZ, UK; Childhood Acute Illness & Nutrition (CHAIN) Network

Tackling of child malnutrition is key to achieving the third Sustainable Development Goal of good health and wellbeing for all. During the past 15 years, improvements have been achieved in overall mortality from acute childhood illnesses such as malaria, diarrhoea, and pneumonia. However, children with impaired growth are still at elevated risk of death from these common infections and are limited in their neurodevelopmental potential. Stunting (ie, low height-for-age) is the commonest form of malnutrition, and the most difficult to prevent or treat.^1^

In *The Lancet Global Health,* Helen Nabwera and colleagues^2^ describe how child growth in a rural African setting has changed during four decades of public health interventions. The investigators analysed data from an unparalleled resource: longitudinal growth monitoring across three Gambian villages spanning 36 years. The long-term presence of a research programme in the community has facilitated free and accessible primary and antenatal care, universal improved water and sanitation, comprehensive immunisation, and systematic screening and treatment for malnutrition. This study therefore represents an unusual setting where the effects of full implementation of international guidelines for health promotion can be observed.

Nabwera and colleagues report a halving of the prevalence of stunting among 2-year-old children over the study period from 1976 to 2012, from 57·1% (95% CI 51·9–62·4) to 30·0% (27·0—33·0). This finding is, in itself, a substantial achievement that exceeds the prediction by Bhutta and colleagues^3^ in 2008 that 99% coverage with combined evidence-based interventions for undernutrition in the 36 highest-burden countries would reduce stunting prevalence among 2-year-old children by 36%. Furthermore, the interventions included in this model—universal supplementation of energy, protein, vitamin A, and zinc, multiple micronutrient supplementation during pregnancy, intermittent preventive treatment for malaria, breastfeeding and complementary feeding support, and hygiene facilitation—together exceed those interventions undertaken in the three study villages.

In this Gambian cohort, progress in stunting and underweight from 1980 onwards stalled. In a separate study of the same three villages from 1950 to 1997, mortality reduction also slowed from 1980 onwards.^4^ These concurrent observations, along with evidence from global meta-analyses demonstrating exponential association of stunting with mortality,^1^ suggest that residual stunting might limit further gains in child survival in this community. We therefore agree with the investigators that the current stunting prevalence of 30% remains unacceptably high. Similar predicaments are seen across sub-Saharan Africa, south Asia, and southeast Asia, where national stunting prevalence commonly exceeds 40%.^1^ Across all developing countries, prevalence of stunting has fallen from 47% in 1980 to 33% in 2000, although this global aggregate masks substantial regional variation.^5^

The persistence of stunting in this Gambian cohort occurred despite a concurrent 80% fall in incidence of malaria, diarrhoea, and bronchiolitis. This disparity supports an emerging consensus that acute illness episodes and nutritional intake alone do not fully account for growth faltering. Chronic, subclinical inflammation is probably a major additional factor. Environmental enteric dysfunction is characterised by morphological and inflammatory changes to the small intestinal lining that are present with varying severity amongst most children in tropical low-income settings. Among the first observations of environmental enteric dysfunction were those made in The Gambia in the late 1980s. These early studies included a 1987–88 infant cohort in the same three villages as described by Nabwera and colleagues.^6^ A combination of increased intestinal permeability and decreased absorptive capacity accounted for 43% of the variability in length gain and 39% of the variability in weight gain. More recently, in 2007–08 high levels of intestinal damage were again noted within children in this same community.^7^ The persistence of environmental enteric dysfunction in this setting—as in other locations—has almost certainly been a major contributor to the stall in stunting reduction. Interventions against environmental enteric dysfunction (eg, probiotics, antibiotics, anthelmintics, and nutritional supplements) have been trialled, with limited or no efficacy on markers of environmental enteric dysfunction or growth.^8^

Global birthweight variability suggests that growth faltering starts before birth and is therefore at least partly affected by prenatal, genetic, and epigenetic exposures.^9^ Across studies, a consistently dominant risk factor for childhood stunting is maternal height.^10^ In this Gambian cohort, only modest improvements in maternal height (by 2·8 cm) and birthweight (by 0·26 weight-for-age *Z* scores) were observed over 36 years (ie, two generations). Interventions to reduce stunting will probably therefore take more than two generations to demonstrate effectiveness.

Both stunting and environmental enteric dysfunction improve with radical shifts in environment, such as migration or local economic development.^11^ This improvement is reflected to some extent by the smoothing of the seasonality of undernutrition reported by Nabwera and colleagues in these three Gambian villages, which they attributed to general improvements in local wealth, food security, and education. Tackling of the residual burden of stunting will therefore likely need multifaceted, community-based interventions. We agree with the investigators’ prediction that basic water, sanitation, and hygiene improvements will be insufficient to further reduce stunting. Much higher-intensity interventions are required—across multiple generations and in combination with economic development—to overcome this barrier to child survival.
